# Evolutionary View of Liver Pathology

**DOI:** 10.1111/eva.70059

**Published:** 2024-12-22

**Authors:** Katalin Dezső, Sándor Paku, Mária‐Manuela Juhász, László Kóbori, Péter Nagy

**Affiliations:** ^1^ Department of Pathology and Experimental Cancer Research Semmelweis University Budapest Hungary; ^2^ Department of Surgery, Transplantation and Gastroenterology Semmelweis University Budapest Hungary

**Keywords:** evolution, hepatitis, hepatocarcinogenesis

## Abstract

Evolutionary medicine emerged in the late twentieth century, integrating principles of natural selection and adaptation with the health sciences. Today, with a rapidly widening gap between the biology of *Homo sapiens* and its environment, maladaptation or maladaptive disorders can be detected in almost all diseases, including liver dysfunction. However, in hepatology, as in most medical specialties, evolutionary considerations are neglected because the majority of the medical community is not familiar with evolutionary principles. The aim of this brief review is to highlight an evolutionary approach that may facilitate understanding various liver diseases.

## 1 Introduction

Evolutionary or Darwinian medicine is a relatively new medical principle. Evolutionary considerations were applied to medical problems during the second half of the twentieth century, but this approach became generally recognized upon the publication of Nesse and Williams' groundbreaking book in 1994, entitled *Why we get sick*: *The new science of Darwinian medicine*. In simple term, the fundamental distinction between evolutionary and conventional branches of medical sciences is that it seeks to answer the “whys” rather than the “hows.” This approach investigates diseases in a broader context.

In evolutionary medicine it is not sufficient to explain cancer as the malfunction of certain genes, but it asks why such genes exist and why they so often fail. The structure and function of the human (and any other organisms') body is not flawless, they are the result of a complex and often subtle network of trade‐offs. The constantly changing conditions require different settings. The consequences of trade‐offs and (frequently inadequate) adaptations represent the natural soil for organ dysfunctions, called “diseases.”

Charles Darwin discovered the basic principles of evolution by studying the natural selection of species, which laws ‐as it later turned out‐ could be applied to practically all biological processes. Evolution is essentially an adaptation based on the random changes of phenotypic, inherited traits.

This groundbreaking observation was made without any knowledge of the mechanisms of inheritance, later discovered to be the nucleic acids containing the hereditary material of information. Thanks to the revolutionary advances in DNA sequencing, an enormous amount of sequence information has resulted in gene‐centered models of most biological processes. One of the missions of evolutionary medicine is to remind us that DNA changes carry a single option, which can only be manifested under the right circumstances (Gatenby, Gillies, and Brown 2011).

In the following sections, we aim to demonstrate through specific examples how the application of an evolutionary perspective can elucidate various facets of hepatic maladies. By using this evolutionary framework, we can achieve a deeper and often different understanding of liver pathology. This enriched comprehension is not merely academic; it holds significant potential to enhance the long‐term recognition, diagnosis, and therapeutic approaches for these disorders. Ultimately, integrating evolutionary insights into hepatology may lead to more effective strategies for managing and treating liver diseases.

## 2 Metabolic Liver Diseases

Metabolic disorders are the most common hepatic disease since the efficient treatment of viral hepatitis. Nonalcoholic fatty liver disease (NAFLD) occurs in nearly 25%–50% of the population in Western countries (Bellentani, Scaglioni, and Marino 2010) and is also common in the developing world. NAFLD is closely related to insulin resistance, diabetes and is often referred to as a component of the metabolic syndrome. Only by exploring its evolutionary origins can we understand this complex metabolic disorder.

Approximately 95% of the history of *Homo sapiens* (Speakman 2013) was characterized by unpredictable food supply, frequent fasting periods and intense physical activity. Survival required extremely flexible metabolic cooperation among several organ systems, with an intense involvement of the liver. Following a large meal, energy was stored as glycogen in the liver and the skeletal muscle and as triglycerides in adipocytes. This process was mainly regulated by the anabolic action of insulin, which stimulated glucose uptake, glycogen synthesis in the liver and muscles, and lipogenesis in adipocytes. Negative regulation of insulin signaling was required to mobilize stored energy during fasting or stress responses. When adipocytes were overloaded with energy surplus that exceeded their normal storage capacity, the adipose tissue became dysfunctional. The extra fat deposited in the liver (hepatic steatosis) is thought to lead to insulin resistance, and subsequently to an increase in hepatic glucose and triglyceride production (Virtue and Vidal‐Puig 2008). Our genes have been positively selected for the efficient use of nutritional energy (often referred to as “thrifty phenotype”) (Hales and Barker 2001). This was useful for hunter‐gatherers for thousands of years, but a disadvantage for members of an affluent society. The result is a serious mismatch between our genetic heritage and our current life style, which is characterized by an abundance of energy‐dense food and low physical activity (Tsatsoulis et al. 2013). Therefore, it is unlikely that any kind of drug therapy for metabolic syndrome/NAFLD would be more effective than significant changes in our sedentary lifestyles and obesogenic diets.

Chronic insidious inflammation is often associated with metabolic syndrome, thus the concept of “metaflation” (Hotamisligil 2006, 2017) was established. Metabolic overload and disrupted metabolic homeostasis may induce a chronic inflammatory response, which in turn can impair systemic metabolic functions, thus triggering a vicious cycle. This process occurs as a combination of steatosis and inflammation in the liver called non‐alcoholic steatohepatitis (NASH). Metaflation has also evolutionary roots. Starvation as well as infection have been a strong selection pressure during our evolutionary history (Barreiro and Quintana‐Murci 2020). The efficient coordination between nutrient sensing and inflammatory signaling is an important adaptive mechanism (Itoh et al. 2022). This is well exemplified by the existence of “fat body” in *Drosophila melanogaster* (Hotamisligil 2006), where the inflammatory system, adipose tissue and liver are organized as one functional unit. The mutual control mechanism between innate immunity, adipose tissue and liver is preserved. Women are somewhat protected from the cardio‐metabolic consequences of obesity, such as NAFLD, at least until menopause (Kautzky‐Willer and Handisurya 2009). This can be explained by a better glycemic control, increased peripheral and hepatic insulin sensitivity compared to men (Tramunt et al. 2020), which is the consequence of a sex dimorphic glucose homeostasis regulation (Mauvais‐Jarvis 2018). The correlation between obesity and metabolic dysfunctions is well documented, however, the causal relationships still need clarification. (Hotamisligil 2017; Saltiel and Olefsky 2017).

The liver, a highly sexually dimorphic organ (Yang et al. 2006) may have a major impact on these mechanisms. This dimorphism is mainly regulated by estrogens (Meda et al. 2020) therefore it disappears after menopause. Estrogen‐driven effects are likely to result from the demand for higher metabolic flexibility in females. This evolutionary flexibility enables the liver to adapt to the changing metabolic needs of the different reproductive stages (pregnancy, lactation) (Della Torre 2020; Della Torre and Maggi 2017).

Conversely, the transcription factor B cell lymphoma 6 (BCL6) masculinizes hepatic gene expression by influencing RNA methylation. The masculine liver gene program provides a substantial survival advantage for male mice during a severe bacterial infection. However, this protection comes at a price in case of dietary excess, when it provokes hepatic steatosis and insulin resistance. Knocking out BCL6 from hepatocytes completely reverses this phenotype (Nikkanen et al. 2022). This straightforward experiment clearly demonstrates the sex‐dependent trade‐off between host defense and metabolism.

The hepatic sexual dimorphism has far reaching consequences, influencing the sex‐dependent incidence of various diseases for example, gall stone disease or even atherosclerosis. Evolutionary considerations regarding metabolic dysfunctions are nicely reviewed by Genné‐Bacon (2014).

## 3 Maintenance and Regeneration of Hepatic Parenchyma

The liver serves as a metabolic interface between the body and variable nutrients and occasionally toxic compounds transported from the gut via the portal vein. This function requires high adaptability, and regarding toxic substances, regenerative capacity. There are excellent, comprehensive reviews on liver regeneration, which discuss all aspects of this very complex biological reaction (Michalopoulos and Bhushan 2021), a synoptic discussion is beyond our scope. This article aims at focusing on the footprints of evolutionary mechanisms.

The two epithelial components of the liver, the hepatocytes and cholangiocytes have common ontogenic origin. They differentiate from hepatoblasts during early embryogenesis, but later, self‐renewal characterizes both populations. No stem cells and their progenies participate in the everyday cell turnover.

Two essentially distinct mechanisms of maintenance/regeneration could be distinguished based on the cellular participants. The basic form of liver regeneration including the physiological cell turnover, response to minor injuries is characterized by cell lineage fidelity. It is able to maintain/ restore the normal hepatic structure and function, which is unequivocally a beneficial biological response. An extreme form of this regeneration has been studied in rodents following 2/3rd partial hepatectomy (Michalopoulos and Bhushan 2021) but human liver can also respond similarly following extensive surgical resection. This mechanism can fail, especially under intense or prolonged insults, and the surviving cells reactivate ancient, ontogenic mechanisms such as transdifferentiation and wound healing (Raven et al. 2017; Wenjuan Pu et al. 2023). The result is a maladaptive regeneration with hepatic dysfunction and further potential complications such as an increased risk of tumor formation. Massive hepatic necrosis with consequent fulminant liver failure is a rare but very severe complication of liver disease with various etiology (Weng et al. 2015). These necrotic livers can regenerate by either of the two regenerative processes mentioned above (Dezső et al. 2020). However, the specific regenerative pathway that becomes activated in response to massive hepatic necrosis is not yet fully understood. This distinction is crucial from a clinical perspective, as the more effective hepatocyte‐driven regeneration could potentially obviate the need for liver transplantation, thereby improving patient outcomes and resource allocation.

### 3.1 Adaptation/Regeneration by Lineage Fidelity

In evolutionary terms, the hepatic parenchyma corresponds to a riparian ecosystem (Alfarouk et al. 2013). Changes in blood flow in the hepatic lobule result in variability in oxygen‐, and metabolites concentrations, which serve as environmental selection forces. Zajicek, Oren, and Weinreb Jr (1985) proposed that under normal conditions, the periportal hepatocytes divide and are responsible for the maintenance of the liver parenchyma (streaming liver hypothesis), while others denied this proposal (Kennedy et al. 1995). Organizing hepatic cell turnover and regeneration proved to be more complex. The evolutionary competition among hepatocytes does not only ensure maintenance, but it also serves as an efficient adaptation. Erick Sandgren et al. (1991) generated albumin‐urokinase transgenic mice. The highly toxic product of the transgene destroyed most of the hepatocytes, but following spontaneous loss of the transgene in some cells by somatic mutation, the “transgenless” hepatocytes were able to repopulate the liver and rescue the animals. Similar processes are not only observed in genetically manipulated animals. The concept of somatic mutation or aneuploidy used to be mostly associated with cancer. However, recently developed large‐scale sequencing technology revealed that these mechanisms are also active in normal, non‐neoplastic tissues (Yizhak et al. 2019). The result is the highly polyclonal nature of the healthy human liver (Brunner et al. 2019). The adaptive role of aneuploidy is well known in Drosophyla and yeast (Rancati et al. 2008). Polyploidization of hepatocytes was also described more, than 100 years ago (Donne et al. 2020). The incidence of polyploidy in hepatocytes depends on age and actual state of the liver. In the normal adult human liver, about 50% of hepatocytes are polyploid and 30%–90% are aneuploid (Duncan 2013). There are several (not mutually exclusive) views regarding the function of hepatic polyploidization: (i) it may represent terminal differentiation, (ii) it can enhance the functional capacity of the liver, (iii) it can serve as a “buffer” against genetic damage and protect the liver from hepatocellular carcinoma (HCC) (Duncan 2020). Polyploid hepatocytes may undergo aberrant cell divisions (Donne et al. 2020). The formation of multipolar mitotic spindle is associated with chromosome segregation defects and consequent aneuploidy. Duncan et al. (2010) demonstrated that hepatocytes can increase or decrease their ploidy. This mechanism, called “ploidy conveyor” can result in enormous genetic heterogeneity. Genetically distinct subsets of hepatocytes are differentially resistant to challenges, and the more resistant cells can preferentially proliferate. The positively selected somatic mutations lead to a liver‐wide increase in fitness (Wang et al. 2023). This fascinating mechanism is essential for maintaining liver function under constantly changing conditions. The conveyor is accelerated under stressful conditions and can promote the adaptation of the liver to adverse circumstances. Adaptation to one peculiar environment affects fitness in another, unrelated environment. The Inherent trade‐off between selected and unselected traits known as antagonistic pleiotropy, can result in fitness gains or losses depending on the environment. The acquisition of aneuploidy can induce a number of adaptive but also maladaptive changes, contributing to unpredictable drug induced liver injuries (DILI) or even to tumorigenesis (see later). Recent studies of other types of tissues suggest that our genome evolves through somatic (epi)mutations from birth or possibly earlier, as we are facing stress and aging (Venkatachalam, Pikarsky, and Ben‐Neriah 2022).

### 3.2 Regeneration by Lineage Switch

If either hepatic epithelial cell compartment is compromised by long lasting or intense injury, a secondary or backup regenerative mechanism is activated. The characteristic feature of this process is that the surviving hepatocytes or cholangiocytes start to behave as facultative progenitor cells and are able to regenerate the other epithelial compartment by lineage switch (Raven et al. 2017; Wenjuan Pu et al. 2023). The expansion of cholangiocyte‐derived progenitor cells is called “oval cell proliferation” in rat and “ductular reaction” in human liver. Interestingly, the oval/ductular cells always expand in a very close relationship with hepatic myofibroblasts, which supply them with the necessary growth factors/cytokines. The myofibroblasts also synthetize abundant extracellular matrix‐ including components of basement membrane‐, required for the expansion of the progenitor cells(Williams et al 2014). The presence of myofibroblasts is essential for this kind of liver regeneration. They provide a “niche” which enables the oval/ductular cells to expand and subsequently differentiate. It can eventuate in particular experimental conditions almost complete restoration of the original liver structure (Dezső et al. 2012). However, mostly when the liver is exposed to sustained injury (viral hepatitis, ALD etc.) the permanent regeneration with the participation of myofibroblasts drives maladaptation with severe adverse consequences. The gradual accumulation of collagenous matrix results in the reconstruction of the hepatic tissue, eventually leading to cirrhosis (Dezső et al. 2022).

The cirrhotic liver consists of hepatocytic nodules separated by fibrotic bundles. There is general agreement that most of the cirrhotic nodules are clonal (Brunner et al. 2019; Gong et al. 2010; Lin et al. 2010). This fact already refers to a selective process, moreover deep sequencing of cirrhotic livers identified recurrent mutations (Zhu et al. 2019). The number and size of mutant clones increased depending on fibrotic stage. Some of these positively selected genes (KMT2D, PKD1) promote hepatocyte fitness, regenerative capacity, but they are not commonly detected in liver cancer. In this respect cirrhosis can be seen as a typical example of evolutionary trade off. Permanent liver injury drives the selection of the hepatocytes with a robust regenerative capacity, but it is also associated with structural alterations, conferring severe hepatic dysfunction. Fibrosis may also be viewed as an evolutionary conserved adaptation mechanism, which can be regarded as a wound healing process in response to epithelial injury. It secures provisional matrix to facilitate regeneration and it may limit the attendant risk of oncogenic transformation (Thannickal et al. 2014). Since no sharing of clonal mutations were found between adjacent nodules, fibrosis can serve as a barrier for spreading the genetic alterations (Brunner et al. 2019).

## 4 Infectious Liver Disease

Infectious diseases cannot be understood without applying evolutionary principles. Evolutionary epidemiology (Ewald 1988) which integrates evolutionary mechanisms, public health and ecology provides a new approach to the study of infectious diseases. Its focus is on the interaction between microbes and host organisms rather than a one‐sided study of infectious agents. Moreover, particular attention is paid to the impact of vaccination and therapeutic interventions (antibiotics/antiviral drugs) on infectious agents responding to medical interventions by adaptive selection.

Liver infections caused by various pathogens are highly prevalent and represent a significant global health concern (Reddy 2020). This review provides a concise overview of the evolutionary aspects of two key types of hepatic infections: biliary tract infections and viral hepatitis. These conditions not only highlight the diversity of infectious agents capable of affecting the liver but also underscore the complex interplay between host defense mechanisms and pathogen evolution, which can influence the pathogenesis, clinical outcomes, and management strategies for these infections.

### 4.1 Infection With Human Liver Flukes

Liver flukes, ( *Clonorchis sinensis* , *Opisthorchis viverrini* , and *Opisthorchis felineus*) the important food‐borne trematodes, represent a major public health problem in large areas of Eastern Europe and Asia. Fluke infection can induce severe hepatobiliary pathology: cholangitis, cholangiofibrosis or eventually even cholangiocarcinoma. Adult flukes do not multiply, but they survive for more than a decade in their human hosts, which has an impact on their fitness.

The evolutionary strategy of the parasites involves the contribution of the adult worms to the host's ability to ensure that the worms have the resources they need.

The helminth infection triggers a pronounced Th2 reaction and expansion of Treg cells (Maizels, Hewitson, and Smith 2012; Mishra et al. 2014). This immunomodulation attenuates responses to non‐helminth antigens, such as allergens and self‐antigens, leading to a reduction in the incidence of allergic and autoimmune diseases in infected individuals (Yazdanbakhsh, van den Biggelaar, and Maizels 2001). A decreased risk of metabolic syndrome (Wiria et al. 2014) and attenuation of atherosclerosis (Magen et al. 2013) has also been reported in the flukes' hosts. The high proportion of asymptomatic carriers also suggests that the immunmodulation may allow fluke infestation and mild pathological alterations in endemic communities (McSorley and Maizels 2012). The outcome of the infection is strongly influenced by the genetic background of the host (Fumagalli et al. 2009). The traditional view that equates disease with infection needs to be complemented by evolutionary thinking which emphasizes the dynamic and context‐dependent nature of the outcome of the infection on a cost–benefit continuum. Given that liver fluke infection may be beneficial depending on the intensity of infection and the genetic background of the host, a more discriminative and individualized treatment strategy should be considered (Echaubard et al. 2016).

### 4.2 Viral Hepatitis

The most common infections are due to hepatotropic viruses (Odenwald and Paul 2022). Vaccination and the revolutionary advances of anti‐viral treatments have drastically changed the outcome of these diseases but at the same time posed new challenges. The two most prevalent forms are hepatitis B and C related diseases.

Hepatitis B viruses can be classified into different genotypes (Lin and Kao 2015). The relationship between genotype and pathological consequences is not entirely understood but there is some coherency for example, HCC is more likely to complicate infections with genotype C, than B (Kao et al. 2000).

The geographical distribution of the genotypes (Okamoto et al. 1988) provides some insight into the historical spreading of HBV for example, the slave trade of African people into America (Paraskevis et al. 2013). However, it cannot be adjusted to the provisional migration pattern of *Homo sapiens* on Earth. Diversification suggests that recent HBVs are the result of multiple cross‐species transmission and recombination. These events might potentially have quite significant public health implications. The endemic infection in nonhuman primates and bats may hamper HBV control in those geographical regions where we share habitats with these animals (Littlejohn, Locarnini, and Yuen 2016).

Administration of recombinant HBsAg vaccine is a success story. It efficiently prevents chronic HBV infection and its complications (e.g., HCC) worldwide (Kane 2003). The vaccination improves the chances of individuals even as a postexposition intervention (infants born from chronically infected mothers or HBV carriers receiving liver transplantation) if it is complemented with HBV‐specific immunoglobulin. However, the failure of antibody co‐administration, mostly due to financial reasons in developing countries, favors the emergence of the vaccine escape mutants (Tabor 2006). Antiviral therapies are the second line of protection against HBV. HBV reverse transcriptase lacks proof reading ability (Orito et al. 1989) leading to high mutation rate and the development of HBV variants resistant to HBV DNA polymerase inhibitors. Antiviral therapy exerts selection pressure and thus paradoxically enhances this process (Fournier and Zoulim 2007).

Unlike HBV, hepatitis C virus (HCV) is a small RNA virus, but also lacks its own RNA‐dependent RNA polymerase proofreading activity. This low‐fidelity, yet extremely efficient replication, results in a large, heterogenous viral population, that is optimal for rapid evolution (Preciado et al. 2014). Besides high mutation rate, HCV molecular evolution is shaped by other mechanisms: genetic bottlenecks, genetic drift and recombination (Kato et al. 1994; Li et al. 2012). Two different levels of HCV evolution can be distinguished (Jackowiak et al. 2014): one that occurs in a single host and the other one within a population. These levels act with different selection pressures, sometimes driving the virus in opposite directions. The result of this complex evolutionary process determines the progression and the outcome of the disease in individual patients. The use of direct‐acting antivirals (DAA) has led to breakthroughs in the treatment of HCV infection. Sustained virological response (SVR) can be achieved in more, than 95% of the patients (Sarrazin 2021). One of the main causes of therapeutic failure is the presence of resistance‐associated viral variants (RAV). They may already be pre‐existing in patients and have been selected by a previous therapy, leading resistance to DAAs (Pawlotsky 2016). The prevalence of naturally occurring RAVs depends on HCV genotypes and varies geographically (Welzel et al. 2017). Evolutionary considerations are important for therapy design for example, pan‐genotypic DAA regimens tend to have higher barriers to resistance, furthermore retreatment with the same regimen is generally not recommended due to a high failure rate (Lawitz et al. 2015).

#### 4.2.1 Liver Cancer

Evolutionary medicine regards cancer as a complex developmental process of somatic clonal evolution. (Scott and Marusyk 2017). Diversification of cancer genotype through mutational and epigenetic alterations is required for adaptability. These changes serve as a substrate on which the forces of selection can act. The cancer phenotype is shaped by its adaptation to specific environments associated with cancer progression. How can the above considerations be applied to liver cancer?

Here we will only discuss the most common primary liver tumor, HCC. Over the last few decades it has become apparent that the oncogenic theory does not solve the mystery of cancer. The mismatch between the pattern of gene mutations and the biology clinical behavior of tumors (Nik‐Zainal et al. 2012) challenges the view that cancer can be conceived by studying gene mutations and a broad new conceptual framework is needed to synthetize new insights.

Interestingly, two of these widespread cancer concepts pay particular attention to viral infections. Hepatitis B and/or C virus infection is implicated in about 80% of liver cancers worldwide (El‐Serag 2012). Ewald and Swain Ewald (2013) put forward the “barrier theory” as a general evolutionary concept of cancer. It assumes that viral infections may enhance the mutation burden, but their ability to remove barriers to cancer development such as cell cycle arrest, apoptosis, telomerase activity and cell adhesion appears to be more important, and the hepatitis B and C viruses have this ability (Ewald and Swain Ewald 2019). According to Shapiro JA one of the proponents of genome chaos theory (2021), viral infections stimulate complex genome remodeling, and genome chaos that can be associated with oncogenesis. Severe genomic alterations resulting from HBV and HCV integration seems to be crucial for hepatocarcinogenesis (Qian et al. 2024; Ye et al. 2023).

The similar transcriptomic landscape and immune profile of HBV‐ and HCV mediated HCCs show a convergent evolutionary pathway of viral hepatocarcinogenesis (Borden et al. 2024).

HCC evolves through a sequential order of genomic and epigenomic alterations. The genomic landscape of primary liver cancers is highly complex (Barcena‐Varela and Lujambio 2021; Ding et al. 2019). In addition to inter‐patient tumoral heterogeneity, multicentric occurrence of HCC is common and intra‐patient inter‐tumoral heterogeneity aggravates therapeutical approaches (Chan et al. 2022). Molecular characterization could distinguish multicentric occurrence and intrahepatic metastases among simultaneously presenting tumor nodules. Multicentric tumors showed a higher degree of heterogeneity, but continuous evolvement of intratumoral metastases can be also observed (Castelli, Pelosi, and Testa 2017). HCC heterogeneity represents an important challenge for genomic classification and contrary to other tumors (e.g., CRC, thyroid) no generally accepted consensus classification is available. It is likely that the complex phenotypes are mainly responsible for the modest survival benefit of personalized therapies (Sorafenib and Lenvanitinib) (Friemel et al. 2014; Kudo et al. 2018). These observations question the reliability of prognostic and predictive factors gained from one histological sample.

The majority of HCCs are formed in cirrhotic livers. Although, as mentioned before, somatic mutations are also present in healthy, normal livers, the mutation burden is higher in cirrhosis, and further increased in tumors. Progressive changes of the (epi)genome are documented during tumorigenesis in liver. Polyploidization has been proposed (Duncan 2020) to protect liver from carcinogenesis. Conversely, while aneuploidy could promote adaptation as well as neoplastic transformation, it can serve as a first hit in‐route to cancer (Duncan 2013). The pattern of mutated genes is also different in tumors for example, TERT, TP53, CTNNB1 mutations are present almost exclusively in cancerous liver tissue (Kim et al. 2019). Also, the types of mutations show some correlations with the etiology of the tumor (Xue et al. 2016). Certainly, there is a close interplay between genetic and epigenetic alterations. In fact, Ding et al. (2019) observed that cirrhotic nodules acquired DNA methylation changes, characteristic of HCC predominate over genome mutations. These epigenetic changes may be the first steps to select growth advantage that is, DNA methylation pattern of cirrhotic livers could reflect the potential upcoming neoplastic transformation.

Genetic injuries are certainly required for tumor development. However, none of the (epi)mutations have absolute fitness value, they can only be estimated in their environment (Scott and Marusyk 2017). Clonal expansion of driver mutations has been found in normal liver tissue without any consequences (Venkatachalam, Pikarsky, and Ben‐Neriah 2022). Multiple observations support the contribution of the microenvironment to liver carcinogenesis. It is most convincingly demonstrated by the simple experiment of Marongiu et al. (2016), where preneoplastic hepatocytes were injected into either young and old syngeneic rats. No visible lesion was detected in any of the young recipients, while tumors were presented in almost all old animals (17/18). Aging is the strongest risk factor for cancer in any tissue (Lemaitre et al. 2020).

In the liver, the complex microenvironmental changes in chronic liver diseases (Hernandez‐Gea et al. 2013) seem to be the most important organ‐specific modifications. Cirrhosis has long been considered a putative premalignant lesion of the liver, yet this lesion is not associated with recurrent somatic/cancer driver mutations (Ding et al. 2019). Instead, the presence of convergent mutations in tumors (P53, TERT, MYC, CTNNB1) suggests that these tumors undergone selection from similar microenvironment. Despite morphological and functional uniformity of cirrhosis there are differences in the microenvironment and the survival of HCC patients, that correlate strongly with the gene expression profile of the surrounding non‐tumor liver tissue (Hoshida et al. 2008). Mutations in genes such as LAMA2, LAMA5, and Coll4A4 support that dysfunction of extracellular matrix pathways may be involved in HCC progression (Xue et al. 2016). The interaction between the tumor and its microenvironment is mutual. HCCs with higher transcriptomic diversity produce more VEGF, which drives microenvironmental reprogramming (Ma et al. 2019). The shaping of microenvironment by the tumor has even been called “cancer niche construction” (Laukien 2021). Notwithstanding, immune mechanisms seem to play the leading role in the tumor—microenvironment interactions. This is a heterogenous, dynamic and also bi‐directional process. Immune pressure potentially drives tumor genomic evolution in response to immune landscapes shaped by the tumor transcriptome (Thorsson et al. 2018). Nguyen et al. (2021) suggest that intratumoral heterogeneity of the immune system is a hallmark of tumor‐immune co‐evolution during HCC progression.

Early‐relapsed HCCs have decreased Tregs increased dendritic cells and CD8^+^ T cells compared to primary tumors (Sun et al. 2021). Zhang et al. (2019) proposed a novel immunophenotypic classification of HCCs. Since this parameter shows less intratumoral heterogeneity, than the mutational landscape, it may improve the decision on the choice of therapy. These and other observations suggest that evaluation and characterization of the microenvironment is becoming a substantial factor in the stratification and treatment of HCC.

## 5 Concluding Remarks

This brief review is not intended to be comprehensive. Only a few selected topics are presented to reveal how the evolutionary approach can be applied to understand hepatic diseases. This list can be further extended by discussing the competitive processes that shape the metastatic cascade, the emergence of immune mediated liver diseases etc. In fact, competition and selection are part of mechanisms that lead to any liver pathology. Yet, evolutionary considerations, are quite scarce in the mainstream hepatology literature. We would like to encourage scientists to examine their results from this perspective.

## Conflicts of Interest

The authors declare no conflicts of interest.

## Data Availability

The authors have nothing to report.

## References

[eva70059-bib-0001] Alfarouk, K. O. , M. E. Ibrahim , R. A. Gatenby , and J. S. Brown . 2013. “Riparian Ecosystems in Human Cancers.” Evolutionary Applications 6: 46–53. 23396634 10.1111/eva.12015 PMC3567470

[eva70059-bib-0002] Barcena‐Varela, M. , and A. Lujambio . 2021. “The Endless Sources of Hepatocellular Carcinoma Heterogeneity.” Cancers (Basel) 13: 2621. 34073538 10.3390/cancers13112621 PMC8198457

[eva70059-bib-0003] Barreiro, L. B. , and L. Quintana‐Murci . 2020. “Evolutionary and Population (Epi)Genetics of Immunity to Infection.” Human Genetics 139: 723–732. 32285198 10.1007/s00439-020-02167-x PMC7285878

[eva70059-bib-0004] Bellentani, S. , F. Scaglioni , and M. Marino . 2010. “Epidemiology of Non‐Alcoholic Fatty Liver Disease.” Digestive Diseases 28: 155–161. 20460905 10.1159/000282080

[eva70059-bib-0005] Borden, E. S. , A. Jorgensen , H. M. Natri , K. T. Hastings , K. H. Buetow , and M. A. Wilson . 2024. “HCV‐ and HBV‐Mediated Liver Cancer Converge on Similar Transcriptomic Landscapes and Immune Profiles.” bioRxiv 6, no. 1: 100373. 10.1016/j.xhgg.2024.100373 PMC11570839 39427232

[eva70059-bib-0006] Brunner, S. F. , N. D. Roberts , L. A. Wylie , et al. 2019. “Somatic Mutations and Clonal Dynamics in Healthy and Cirrhotic Human Liver.” Nature 574: 538–542. 31645727 10.1038/s41586-019-1670-9 PMC6837891

[eva70059-bib-0007] Castelli, G. , E. Pelosi , and U. Testa . 2017. “Liver Cancer: Molecular Characterization, Clonal Evolution and Cancer Stem Cells.” Cancers (Basel) 9: 127. 28930164 10.3390/cancers9090127 PMC5615342

[eva70059-bib-0008] Chan, L. K. , Y. M. Tsui , D. Wai‐Hung , and I. Oi‐Lin Ng . 2022. “Cellular Heterogeneity and Plasticity in Liver Cancer.” Seminars in Cancer Biology 82: 134–149. 33647386 10.1016/j.semcancer.2021.02.015

[eva70059-bib-0009] Della Torre, S. 2020. “Non‐alcoholic Fatty Liver Disease as a Canonical Example of Metabolic Inflammatory‐Based Liver Disease Showing a Sex‐Specific Prevalence: Relevance of Estrogen Signaling.” Front Endocrinol (Lausanne) 11: 572490. 33071979 10.3389/fendo.2020.572490 PMC7531579

[eva70059-bib-0010] Della Torre, S. , and A. Maggi . 2017. “Sex Differences: A Resultant of an Evolutionary Pressure?” Cell Metabolism 25: 499–505. 28190772 10.1016/j.cmet.2017.01.006

[eva70059-bib-0011] Dezső, K. , P. Nagy , and S. Paku . 2020. “Human Liver Regeneration Following Massive Hepatic Necrosis: Two Distinct Patterns.” Journal of Gastroenterology and Hepatology 35: 124–134. 31090096 10.1111/jgh.14721

[eva70059-bib-0012] Dezső, K. , S. Paku , L. Kóbori , S. S. Thorgeirsson , and P. Nagy . 2022. “What Makes Cirrhosis Irreversible? Considerations on Structural Changes.” Frontiers in Medicine 9: 876293. 35572980 10.3389/fmed.2022.876293 PMC9091510

[eva70059-bib-0013] Dezső, K. , V. Papp , E. Bugyik , et al. 2012. “Structural Analysis of Oval‐Cell Mediated Liver Regeneration in Rats.” Hepatology 56: 1457–1467. 22419534 10.1002/hep.25713

[eva70059-bib-0014] Ding, X. , M. He , A. W. H. Chan , et al. 2019. “Genomic and Epigenomic Features of Primary and Recurrent Hepatocellular Carcinomas.” Gastroenterology 157: 1630–1645. 31560893 10.1053/j.gastro.2019.09.005

[eva70059-bib-0015] Donne, R. , M. Saroul‐Aïnama , P. Cordier , S. Celton‐Morizur , and C. Desdouets . 2020. “Polyploidy in Liver Development, Homeostasis and Disease.” Nature Reviews. Gastroenterology & Hepatology 17: 391–405. 32242122 10.1038/s41575-020-0284-x

[eva70059-bib-0016] Duncan, A. W. 2013. “Aneuploidy, Polyploidy and Ploidy Reversal in the Liver.” Seminars in Cell & Developmental Biology 24: 347–356. 23333793 10.1016/j.semcdb.2013.01.003

[eva70059-bib-0017] Duncan, A. W. 2020. “Hepatocyte Ploidy Modulation in Liver Cancer.” EMBO Reports 21, no. 12: e51922. 33237586 10.15252/embr.202051922 PMC7726811

[eva70059-bib-0018] Duncan, A. W. , M. H. Taylor , R. D. Hickey , et al. 2010. “The Ploidy‐Conveyor of Mature Hepatocytes as a Source of Genetic Variation.” Nature 467: 707–710. 20861837 10.1038/nature09414 PMC2967727

[eva70059-bib-0019] Echaubard, P. , B. Sripa , F. F. Mallory , and B. A. Wilcox . 2016. “The Role of Evolutionary Biology in Research and Control of Liver Flukes in Southeast Asia.” Infection, Genetics and Evolution 43: 381–397. 10.1016/j.meegid.2016.05.019 PMC4971136 27197053

[eva70059-bib-0020] El‐Serag, H. B. 2012. “Epidemiology of Viral Hepatitis and Hepatocellular Carcinoma.” Gastroenterology 142: 1264–1273. 22537432 10.1053/j.gastro.2011.12.061 PMC3338949

[eva70059-bib-0021] Ewald, P. W. 1988. “Cultural Vectors, Virulence and the Emergence of Evolutionary Epidemiology.” In Oxford Surveys in Evolutionary Biology, vol. 5, 215–245. Oxford, UK: Oxford University Press.

[eva70059-bib-0022] Ewald, P. W. , and H. A. Swain Ewald . 2013. “Toward a General Evolutionary Theory of Oncogenesis.” Evolutionary Applications 6: 70–81. 23396676 10.1111/eva.12023 PMC3567472

[eva70059-bib-0023] Ewald, P. W. , and H. A. Swain Ewald . 2019. “The Scope of Viral Causation of Human Cancers: Interpreting Virus Density From an Evolutionary Perspective.” Philosophical Transactions of the Royal Society of London. Series B, Biological Sciences 374, no. 1773: 20180304. 30955500 10.1098/rstb.2018.0304 PMC6501912

[eva70059-bib-0024] Fournier, C. , and F. Zoulim . 2007. “Antiviral Therapy of Chronic Hepatitis B: Prevention of Drug Resistance.” Clinics in Liver Disease 11: 869–892. 17981233 10.1016/j.cld.2007.08.013

[eva70059-bib-0025] Friemel, J. , M. Rechsteiner , L. Frick , et al. 2014. “Intratumor Heterogeneity in Hepatocellular Carcinoma.” Clinical Cancer Research 21: 1951–1961. 25248380 10.1158/1078-0432.CCR-14-0122

[eva70059-bib-0026] Fumagalli, M. , U. Pozzoli , R. Cagliani , et al. 2009. “Parasites Represent a Major Selective Force for Interleukin Genes and Shape the Genetic Predisposition to Autoimmune Conditions.” Journal of Experimental Medicine 206, no. 6: 1395–1408. 19468064 10.1084/jem.20082779 PMC2715056

[eva70059-bib-0027] Gatenby, R. A. , R. J. Gillies , and J. S. Brown . 2011. “Of Cancer and Cave Fish.” Nature Reviews. Cancer 11, no. 4: 237–238. 10.1038/nrc3036 PMC3971416 21548400

[eva70059-bib-0028] Genné‐Bacon, E. A. 2014. “Thinking Evolutionarily About Obesity.” Yale Journal of Biology and Medicine 87, no. 2: 99–112. 24910556 PMC4031802

[eva70059-bib-0029] Gong, L. , Y. H. Li , Q. Su , X. Chu , and W. Zhang . 2010. “Clonality of Nodular Lesions in Liver Cirrhosis and Chromosomal Abnormalities in Monoclonal Nodules of Altered Hepatocytes.” Histopathology 56: 589–599. 20459569 10.1111/j.1365-2559.2010.03523.x

[eva70059-bib-0030] Hales, C. N. , and D. J. Barker . 2001. “The Thrifty Phenotype Hypothesis.” British Medical Bulletin 60: 5–20. 11809615 10.1093/bmb/60.1.5

[eva70059-bib-0031] Hernandez‐Gea, V. , S. Toffanin , S. L. Friedman , and J. M. Llovet . 2013. “Role of the Microenvironment in the Pathogenesis and Treatment of Hepatocellular Carcinoma.” Gastroenterology 144, no. 3: 512–527. 23313965 10.1053/j.gastro.2013.01.002 PMC3578068

[eva70059-bib-0032] Hoshida, Y. , A. Villanueva , M. Kobayashi , et al. 2008. “Gene Expression in Fixed Tissues and Outcome in Hepatocellular Carcinoma.” New England Journal of Medicine 359, no. 19: 1995–2004. 18923165 10.1056/NEJMoa0804525 PMC2963075

[eva70059-bib-0033] Hotamisligil, G. S. 2006. “Inflammation and Metabolic Disorders.” Nature 444, no. 7121: 860–867. 17167474 10.1038/nature05485

[eva70059-bib-0034] Hotamisligil, G. S. 2017. “Inflammation, Metaflammation and Immunometabolic Disorders.” Nature 542, no. 7640: 177–185. 28179656 10.1038/nature21363

[eva70059-bib-0035] Itoh, H. , M. Ueda , M. Suzuki , and Y. Kohmura‐Kobayashi . 2022. “Developmental Origins of Metaflammation; A Bridge to the Future Between the DOHaD Theory and Evolutionary Biology.” Front Endocrinol (Lausanne) 13: 839436. 35185805 10.3389/fendo.2022.839436 PMC8850935

[eva70059-bib-0036] Jackowiak, P. , K. Kuls , L. Budzko , A. Mania , and M. Figlerowicz . 2014. “Phylogeny and Molecular Evolution of Hepatitis C Virus Infection.” Infection, Genetics and Evolution 21: 67–82. 10.1016/j.meegid.2013.10.021 24200590

[eva70059-bib-0037] Kane, M. A. 2003. “Global Control of Primary Hepatocellular Carcinoma With Hepatitis B Vaccine: The Contributions of Research in Taiwan.” Cancer Epidemiology, Biomarkers & Prevention 12, no. 1: 2–3. 12540496

[eva70059-bib-0038] Kao, J. H. , P. J. Chen , M. Y. Lai , and D. S. Chen . 2000. “Hepatitis B Genotypes Correlate With Clinical Outcomes in Patients With Chronic Hepatitis B.” Gastroenterology 118, no. 3: 554–559. 10702206 10.1016/s0016-5085(00)70261-7

[eva70059-bib-0039] Kato, N. , Y. Ootsuyama , H. Sekiya , et al. 1994. “Genetic Drift in Hypervariable Region 1 of the Viral Genome in Persistent Hepatitis C Virus Infection.” Journal of Virology 68, no. 8: 4776–4784. 7518526 10.1128/jvi.68.8.4776-4784.1994 PMC236417

[eva70059-bib-0040] Kautzky‐Willer, A. , and A. Handisurya . 2009. “Metabolic Diseases and Associated Complications: Sex and Gender Matter!” European Journal of Clinical Investigation 39, no. 8: 631–648. 19496803 10.1111/j.1365-2362.2009.02161.x

[eva70059-bib-0041] Kennedy, S. , S. Rettinger , M. W. Flye , and K. P. Ponder . 1995. “Experiments in Transgenic Mice Show That Hepatocytes Are the Source for Postnatal Liver Growth and Do Not Stream.” Hepatology 22, no. 1: 160–168. 7541385

[eva70059-bib-0042] Kim, S. K. , H. Takeda , A. Takai , et al. 2019. “Comprehensive Analysis of Genetic Aberrations Linked to Tumorigenesis in Regenerative Nodules of Liver Cirrhosis.” Journal of Gastroenterology 54, no. 7: 628–640. 30756187 10.1007/s00535-019-01555-z

[eva70059-bib-0043] Kudo, M. , R. S. Finn , S. Qin , et al. 2018. “Lenvatinib Versus Sorafenib in First‐Line Treatment of Patients With Unresectable Hepatocellular Carcinoma: A Randomised Phase 3 Non‐inferiority Trial.” Lancet 391, no. 10126: 1163–1173. 29433850 10.1016/S0140-6736(18)30207-1

[eva70059-bib-0044] Laukien, F. H. 2021. “The Evolution of Evolutionary Processes in Organismal and Cancer Evolution.” Progress in Biophysics and Molecular Biology 165: 43–48. 34509467 10.1016/j.pbiomolbio.2021.08.008

[eva70059-bib-0045] Lawitz, E. , S. Flamm , J. Yang , et al. 2015. “Retreatment of Patients Who Failed 8 or 12 Weeks of Ledipasvir/Sofosbuvir–Based Regimens With Ledipavir/Sofosuvir for 24 Weeks.” Journal of Hepatology 62: S192.

[eva70059-bib-0046] Lemaitre, J. F. , S. Pavard , M. Giraudeau , et al. 2020. “Eco‐Evolutionary Perspectives of the Dynamic Relationships Linking Senescence and Cancer.” Functional Ecology 34: 141–152.

[eva70059-bib-0047] Li, H. , M. B. Stoddard , S. Wang , et al. 2012. “Elucidation of Hepatitis C Virus Transmission and Early Diversification by Single Genome Sequencing.” PLoS Pathogens 8, no. 8: e1002880. 22927816 10.1371/journal.ppat.1002880 PMC3426529

[eva70059-bib-0048] Lin, C. L. , and J. H. Kao . 2015. “Hepatitis B Virus Genotypes and Variants.” Cold Spring Harbor Perspectives in Medicine 5, no. 5: a021436. 25934462 10.1101/cshperspect.a021436 PMC4448583

[eva70059-bib-0049] Lin, W. R. , S. N. Lim , S. A. McDonald , et al. 2010. “The Histogenesis of Regenerative Nodules in Human Liver Cirrhosis.” Hepatology 51, no. 3: 1017–1026. 20198634 10.1002/hep.23483

[eva70059-bib-0050] Littlejohn, M. , S. Locarnini , and L. Yuen . 2016. “Origins and Evolution of Hepatitis B Virus and Hepatitis D Virus.” Cold Spring Harbor Perspectives in Medicine 6, no. 1: a021360. 26729756 10.1101/cshperspect.a021360 PMC4691804

[eva70059-bib-0051] Ma, L. , M. O. Hernandez , Y. Zhao , et al. 2019. “Tumor Cell Biodiversity Drives Microenvironmental Reprogramming in Liver Cancer.” Cancer Cell 36, no. 4: 418–430. 31588021 10.1016/j.ccell.2019.08.007 PMC6801104

[eva70059-bib-0052] Magen, E. , V. Bychkov , A. Ginovker , and E. Kashuba . 2013. “Chronic Opisthorchis Felineus Infection Attenuates Atherosclerosis–An Autopsy Study.” International Journal for Parasitology 43, no. 10: 819–824. 23792298 10.1016/j.ijpara.2013.04.008

[eva70059-bib-0053] Maizels, R. M. , J. P. Hewitson , and K. A. Smith . 2012. “Susceptibility and Immunity to Helminth Parasites.” Current Opinion in Immunology 24, no. 4: 459–466. 22795966 10.1016/j.coi.2012.06.003 PMC3437973

[eva70059-bib-0054] Marongiu, F. , M. P. Serra , S. Doratiotto , et al. 2016. “Aging Promotes Neoplastic Disease Through Effects on the Tissue Microenvironment.” Aging (Albany NY) 8, no. 12: 3390–3399. 27929382 10.18632/aging.101128 PMC5270675

[eva70059-bib-0055] Mauvais‐Jarvis, F. 2018. “Gender Differences in Glucose Homeostasis and Diabetes.” Physiology & Behavior 187: 20–23. 28843891 10.1016/j.physbeh.2017.08.016 PMC5826763

[eva70059-bib-0056] McSorley, H. J. , and R. M. Maizels . 2012. “Helminth Infections and Host Immune Regulation.” Clinical Microbiology Reviews 25, no. 4: 585–608. 23034321 10.1128/CMR.05040-11 PMC3485755

[eva70059-bib-0057] Meda, C. , M. Barone , N. Mitro , et al. 2020. “Hepatic ERα Accounts for Sex Differences in the Ability to Cope With an Excess of Dietary Lipids.” Molecular Metabolism 32: 97–108. 32029233 10.1016/j.molmet.2019.12.009 PMC6957843

[eva70059-bib-0058] Michalopoulos, G. K. , and B. Bhushan . 2021. “Liver Regeneration: Biological and Pathological Mechanisms and Implications.” Nature Reviews. Gastroenterology & Hepatology 18, no. 1: 40–55. 32764740 10.1038/s41575-020-0342-4

[eva70059-bib-0059] Mishra, P. K. , M. Palma , D. Bleich , P. Loke , and W. C. Gause . 2014. “Systemic Impact of Intestinal Helminth Infections.” Mucosal Immunology 7, no. 4: 753–762. 24736234 10.1038/mi.2014.23

[eva70059-bib-0060] Nguyen, P. H. D. , S. Ma , C. Z. J. Phua , et al. 2021. “Intratumoural Immune Heterogeneity as a Hallmark of Tumour Evolution and Progression in Hepatocellular Carcinoma.” Nature Communications 12, no. 1: 227. 10.1038/s41467-020-20171-7 PMC7801667 33431814

[eva70059-bib-0061] Nikkanen, J. , Y. A. Leong , W. C. Krause , et al. 2022. “An Evolutionary Trade‐Off Between Host Immunity and Metabolism Drives Fatty Liver in Male Mice.” Science 378, no. 6617: 290–295. 36264814 10.1126/science.abn9886 PMC9870047

[eva70059-bib-0062] Nik‐Zainal, S. , P. Van Loo , D. C. Wedge , et al. 2012. “The Life History of 21 Breast Cancers.” Cell 149: 994–1007. 22608083 10.1016/j.cell.2012.04.023 PMC3428864

[eva70059-bib-0063] Odenwald, M. A. , and S. Paul . 2022. “Viral Hepatitis: Past Present, Future.” World Journal of Gastroenterology 14: 1405–1429. 10.3748/wjg.v28.i14.1405 PMC9048475 35582678

[eva70059-bib-0064] Okamoto, H. , F. Tsuda , H. Sakugawa , et al. 1988. “Typing Hepatitis B Virus by Homology in Nucleotide Sequence: Comparison of Surface Antigen Subtypes.” Journal of General Virology 69, no. Pt 10: 2575–2583. 3171552 10.1099/0022-1317-69-10-2575

[eva70059-bib-0065] Orito, E. , M. Mizokami , Y. Ina , et al. 1989. “Host‐Independent Evolution and a Genetic Classification of the Hepadnavirus Family Based on Nucleotide Sequences.” Proceedings of the National Academy of Sciences of the United States of America 86, no. 18: 7059–7062. 2780562 10.1073/pnas.86.18.7059 PMC297993

[eva70059-bib-0066] Paraskevis, D. , G. Magiorkinis , E. Magiorkinis , et al. 2013. “Dating the Origin and Dispersal of Hepatitis B Virus Infection in Humans and Primates.” Hepatology 57, no. 3: 908–916. 22987324 10.1002/hep.26079

[eva70059-bib-0067] Pawlotsky, J. M. 2016. “Hepatitis C Virus Resistance to Direct Acting Antiviral Drugs in Interferon Free Regimens.” Gastroenterology 151, no. 1: 70–86. 27080301 10.1053/j.gastro.2016.04.003

[eva70059-bib-0068] Preciado, M. V. , P. Valva , A. Escobar‐Gutierrez , et al. 2014. “Hepatitis C Virus Molecular Evolution: Transmission, Disease Progression and Antiviral Therapy.” World Journal of Gastroenterology 20, no. 43: 15992–16013. 25473152 10.3748/wjg.v20.i43.15992 PMC4239486

[eva70059-bib-0069] Pu, W. , H. Zhu , M. Zhang , et al. 2023. “Bipotent Transitional Liver Progenitor Cells Contribute to Liver Regeneration.” Nature Genetics 55, no. 4: 651–664. 36914834 10.1038/s41588-023-01335-9 PMC10101857

[eva70059-bib-0070] Qian, Z. , J. Liang , R. Huang , et al. 2024. “HBV Integrations Reshaping Genomic Structures Promote Hepatocellular Carcinoma.” Gut 73, no. 7: 1169–1182. 38395437 10.1136/gutjnl-2023-330414 PMC11187386

[eva70059-bib-0071] Rancati, G. , N. Pavelka , B. Fleharty , et al. 2008. “Aneuploidy Underlies Rapid Adaptive Evolution of Yeast Cells Deprived of a Conserved Cytokinesis Motor.” Cell 135, no. 5: 879–893. 19041751 10.1016/j.cell.2008.09.039 PMC2776776

[eva70059-bib-0072] Raven, A. , W. Y. Lu , T. Y. Man , et al. 2017. “Cholangiocytes Act as Facultative Liver Stem Cells During Impaired Hepatocyte Regeneration.” Nature 547, no. 7663: 350–354. 28700576 10.1038/nature23015 PMC5522613

[eva70059-bib-0073] Reddy, K. R. 2020. “Infections of the Liver and Biliary System.” Gastroenterology Clinics of North America 49, no. 2: xv–xvi. 10.1016/j.gtc.2020.01.015 32389371

[eva70059-bib-0074] Saltiel, A. R. , and J. M. Olefsky . 2017. “Inflammatory Mechanisms Linking Obesity and Metabolic Disease.” Journal of Clinical Investigation 127, no. 1: 1–4. 28045402 10.1172/JCI92035 PMC5199709

[eva70059-bib-0075] Sandgren, E. P. , R. D. Palmiter , J. L. Heckel , C. C. Daugherty , R. L. Brinster , and J. L. Degen . 1991. “Complete Hepatic Regeneration After Somatic Deletion of an Albumin‐Plasminogen Activator Transgene.” Cell 66, no. 2: 245–256. 1713128 10.1016/0092-8674(91)90615-6

[eva70059-bib-0076] Sarrazin, C. 2021. “Treatment Failure With DAA Therapy: Importance of Resistance.” Journal of Hepatology 74, no. 6: 1472–1482. 33716089 10.1016/j.jhep.2021.03.004

[eva70059-bib-0077] Scott, J. , and A. Marusyk . 2017. “Somatic Clonal Evolution: A Selection‐Centric Perspective.” Biochimica Et Biophysica Acta. Reviews on Cancer 1867, no. 2: 139–150. 28161395 10.1016/j.bbcan.2017.01.006

[eva70059-bib-0078] Shapiro, J. A. 2021. “How Chaotic Is Genome Chaos?” Cancers (Basel) 13, no. 6: 1358. 33802828 10.3390/cancers13061358 PMC8002653

[eva70059-bib-0079] Speakman, J. R. 2013. “Evolutionary Perspectives on the Obesity Epidemic: Adaptive, Maladaptive, and Neutral Viewpoints.” Annual Review of Nutrition 33: 289–317. 10.1146/annurev-nutr-071811-150711 23862645

[eva70059-bib-0080] Sun, Y. , L. Wu , Y. Zhong , et al. 2021. “Single‐Cell Landscape of the Ecosystem in Early‐Relapse Hepatocellular Carcinoma.” Cell 184, no. 2: 404–421.e16. 33357445 10.1016/j.cell.2020.11.041

[eva70059-bib-0081] Tabor, E. 2006. “Infections by Hepatitis B Surface Antigen Gene Mutants in Europe and North America.” Journal of Medical Virology 78, no. Suppl 1: S47. 10.1002/jmv.20606 16622882

[eva70059-bib-0082] Thannickal, V. J. , Y. Zhou , A. Gaggar , and S. R. Duncan . 2014. “Fibrosis: Ultimate and Proximate Causes.” Journal of Clinical Investigation 124, no. 11: 4673–4677. 25365073 10.1172/JCI74368 PMC4347226

[eva70059-bib-0083] Thorsson, V. , D. L. Gibbs , S. D. Brown , et al. 2018. “The Immune Landscape of Cancer.” Immunity 48, no. 4: 812–830. 29628290 10.1016/j.immuni.2018.03.023 PMC5982584

[eva70059-bib-0084] Tramunt, B. , S. Smati , N. Grandgeorge , et al. 2020. “Sex Differences in Metabolic Regulation and Diabetes Susceptibility.” Diabetologia 63, no. 3: 453–461. 31754750 10.1007/s00125-019-05040-3 PMC6997275

[eva70059-bib-0085] Tsatsoulis, A. , M. D. Mantzaris , S. Bellou , and M. Andrikoula . 2013. “Insulin Resistance: An Adaptive Mechanism Becomes Maladaptive in the Current Environment–An Evolutionary Perspective.” Metabolism 62, no. 5: 622–633. 23260798 10.1016/j.metabol.2012.11.004

[eva70059-bib-0086] Venkatachalam, A. , E. Pikarsky , and Y. Ben‐Neriah . 2022. “Putative Homeostatic Role of Cancer Driver Mutations.” Trends in Cell Biology 32, no. 1: 8–17. 34373150 10.1016/j.tcb.2021.07.002

[eva70059-bib-0087] Virtue, S. , and A. Vidal‐Puig . 2008. “It's Not How Fat You Are, it's What You Do With It That Counts.” PLoS O Biologico 6, no. 9: e237. 10.1371/journal.pbio.0060237 PMC2553843 18816166

[eva70059-bib-0088] Wang, Z. , S. Zhu , Y. Jia , et al. 2023. “Positive Selection of Somatically Mutated Clones Identifies Adaptive Pathways in Metabolic Liver Disease.” Cell 186, no. 9: 1968–1984.e20. 37040760 10.1016/j.cell.2023.03.014 PMC10321862

[eva70059-bib-0089] Welzel, T. M. , N. Bhardwaj , C. Hedskog , et al. 2017. “Global Epidemiology of HCV Subtypes and Resistance‐Associated Substitutions Evaluated by Sequencing‐Based Subtype Analyses.” Journal of Hepatology 67, no. 2: 224–236. 28343981 10.1016/j.jhep.2017.03.014

[eva70059-bib-0090] Weng, H. L. , X. Cai , X. Yuan , et al. 2015. “Two Sides of One Coin: Massive Hepatic Necrosis and Progenitor Cell‐Mediated Regeneration in Acute Liver Failure.” Frontiers in Physiology 6: 178. 26136687 10.3389/fphys.2015.00178 PMC4468385

[eva70059-bib-0091] Williams, M. J. , A. D. Clouston , and S. J. Forbes . 2014. “Links Between Hepatic Fibrosis, Ductular Reaction, and Progenitor Cell Expansion.” Gastroenterology 146, no. 2: 349–356. 24315991 10.1053/j.gastro.2013.11.034

[eva70059-bib-0092] Wiria, A. E. , E. Sartono , T. Supali , and M. Yazdanbakhsh . 2014. “Helminth Infections, Type‐2 Immune Response, and Metabolic Syndrome.” PLoS Pathogens 10, no. 7: e1004140. 24992724 10.1371/journal.ppat.1004140 PMC4081794

[eva70059-bib-0093] Xue, R. , R. Li , H. Guo , et al. 2016. “Variable Intra‐Tumor Genomic Heterogeneity of Multiple Lesions in Patients With Hepatocellular Carcinoma.” Gastroenterology 150, no. 4: 998–1008. 26752112 10.1053/j.gastro.2015.12.033

[eva70059-bib-0094] Yang, X. , E. E. Schadt , S. Wang , et al. 2006. “Tissue‐Specific Expression and Regulation of Sexually Dimorphic Genes in Mice.” Genome Research 16, no. 8: 995–1004. 16825664 10.1101/gr.5217506 PMC1524872

[eva70059-bib-0095] Yazdanbakhsh, M. , A. van den Biggelaar , and R. M. Maizels . 2001. “Th2 Responses Without Atopy: Immunoregulation in Chronic Helminth Infections and Reduced Allergic Disease.” Trends in Immunology 22, no. 7: 372–377. 11429321 10.1016/s1471-4906(01)01958-5

[eva70059-bib-0096] Ye, R. , A. Wang , B. Bu , et al. 2023. “Viral Oncogenes, Viruses, and Cancer: A Third‐Generation Sequencing Perspective on Viral Integration Into the Human Genome.” Frontiers in Oncology 13: 1333812. 38188304 10.3389/fonc.2023.1333812 PMC10768168

[eva70059-bib-0097] Yizhak, K. , F. Aguet , J. Kim , et al. 2019. “RNA Sequence Analysis Reveals Macroscopic Somatic Clonal Expansion Across Normal Tissues.” Science 364, no. 6444: eaaw0726. 31171663 10.1126/science.aaw0726 PMC7350423

[eva70059-bib-0098] Zajicek, G. , R. Oren , and M. Weinreb Jr. 1985. “The Streaming Liver.” Liver 5, no. 6: 293–300. 4088003 10.1111/j.1600-0676.1985.tb00252.x

[eva70059-bib-0099] Zhang, Q. , Y. Lou , J. Yang , et al. 2019. “Integrated Multiomic Analysis Reveals Comprehensive Tumour Heterogeneity and Novel Immunophenotypic Classification in Hepatocellular Carcinomas.” Gut 68, no. 11: 2019–2031. 31227589 10.1136/gutjnl-2019-318912 PMC6839802

[eva70059-bib-0100] Zhu, M. , T. Lu , Y. Jia , et al. 2019. “Somatic Mutations Increase Hepatic Clonal Fitness and Regeneration in Chronic Liver Disease.” Cell 177, no. 3: 608–621.e12. 30955891 10.1016/j.cell.2019.03.026 PMC6519461

[eva70059-bib-0101] Zoulim, F. , M. Buti , and A. S. Lok . 2007. “Antiviral‐Resistant Hepatitis B Virus: Can We Prevent This Monster From Growing?” Journal of Viral Hepatitis 14, no. Suppl 1: 29–36. 10.1111/j.1365-2893.2007.00915.x 17958640

